# Rabies1Presented at the Fourteenth Annual Meeting of the Iowa State Veterinary Medical Association, Des Moines, Iowa, February 11 and 12, 1902.

**Published:** 1902-04

**Authors:** D. E. Baughman

**Affiliations:** Fort Dodge, Iowa


### RABIES.[1]

By D. E. Baughman, M.D.C.,
FORT DODGE, IOWA.

At the earnest request of the President and Secretary I have endeavored to prepare a paper on "Rabies"—a disease that has interested me the past season, and I hope I will be successful in interesting the other members of the Association.

There has, perhaps, never been a time when it was more important than at the present for the veterinary profession to have a clear appreciation of the subject of rabies in animals and man. There was a time in the period of the profession's existence when there was excuse for differences of opinion in regard to this disease. It was a time when we depended solely upon clinical observation of accidental cases, and when the conclusions were founded upon imperfect evidence; but that time is past. To-day we have a science resting upon an experimental basis. Facts and conclusions have been established just as rigorously and as solidly as in other departments of medical science.

As members of a learned profession it is our duty to know what has been accomplished by scientific investigators of rabies, and particularly is this duty incumbent upon those who attempt to teach other members of the profession or the laity as to the facts in the case. We have reached a point where the intelligence and scientific knowledge of the veterinary profession are liable to be unjustly questioned because of a few mistakes of misguided individuals who persist in reiterating beliefs which were never held by a majority of the profession and were discarded and disproved years ago. It appears to the writer simply astounding that there are educated men, much less physicians and veterinarians, who will still doubt the contagiousness of this disease, which was known and described in ancient times, and which for a century has been the subject of

[1] Presented at the Fourteenth Annual Meeting of the Iowa State Veterinary Medical Association, Des Moines, Iowa, February 11 and 12, 1902.

experimental investigation by very able men who have occupied their minds with pathological questions. Nevertheless, it is a fact that the sanitarian of to-day who tries to control rabies meets the same kind of argument which was used to embarrass his profession centuries ago. These arguments are most industriously circulated by the so-called humane societies, which oppose all conclusions based upon experiments with animals, and which imagine that they are doing a great work for dogs and cats by casting discredit upon science, even though by so doing they perpetuate this terrible disease, of which dogs and cats are the principal victims.

Aristotle described rabies 400 years B.C., and indicated its transmissibility in these terms: "Dogs suffer from hydrophobia, which provokes in them a state of madness. All animals bitten by dogs affected become rabid in the end." From that time to the present we have clear accounts of the disease existing through every age and provoking horror and fear in many centuries. It was always said to be caused by the bite of an animal, which animal was generally alleged to be rabid. It was almost universally described as fatal in man and animal.

Aristotle admitted that the disease was fatal to dogs and every other creature which they bite except man. This early mistake in regard to the immunity of man has been handed across the centuries, and is still repeated on every hand by those who oppose measures for the prevention of the disease. It may be freely admitted, therefore, that there have probably been many at all ages who have doubted the existence of the disease, both in mankind and in animal; that numerous articles and books have been written to prove that the disease called rabies is not contagious, and that the supposed rabies of man is lyssophobia, a nervous affection brought on by fear and excitement.

The medical profession as a whole, however, has always recognized the existence of such a disease as rabies in man, and the veterinary profession has from its foundation recognized its existence and the contagiousness of the disease. Its schools from the earliest to the latest have constantly taught this doctrine, and the present text-books are all unanimous on the subject.

The doubts raised from time to time concerning rabies and its characteristics have been met by scientific experiments.

Zinka, in 1804, announced that he had inoculated a dog, a rabbit, and a cock with saliva from a rabid dog, taking the saliva with a brush from the animal soon after its death and spreading it over superficial wounds of the inoculated animals. The dog was inoculated on the anterior limb, and showed prodromic symptoms on the eighth day and was rabid on the ninth day. The rabbit was rabid on the fourteenth day and the cock on the eleventh day. This experiment, early in the eighteenth century, proved that the disease of the dog called rabies was communicable by inoculation to the dog, the rabbit, and the fowl. It proved it to be a specific disease and that the virus existed in the saliva.

Reifershield, in 1813, records an experiment in which several dogs were inoculated, part with fluid and part with dried saliva from a rabid dog. These became affected with rabies after eight to ten days.

Bendt, in 1822, inoculated four wethers with saliva from the mouth of an ox which had died of rabies. All of these sheep contracted the disease, the period of inoculation being from twenty-two to thirty-one days.

In Hertwig's experiments he produced rabies by inoculation in 37 per cent. of cases. Renault produced it in 67 per cent.

Haubner gives an average of 40 per cent. of cases in rabies which were contracted through bites. Bollinger states that in man infection occurs in from 8 to 47 per cent. of bites. Pasteur says the proportion varies from 16 to 80 per cent. When cauterization is not performed it reaches 83 per cent. Bouley found that 90 per cent. suffered after bites on the face; 63 per cent. after bites on the hands; 24 per cent. after bites on the arms; 77 per cent. after bites on the legs, and 63 per cent. after bites on the body. The susceptibility of sheep is known to be the slightest, as the teeth of the biting animal are likely to be cleaned on the wool. Much, however, depends upon the stage of the disease and the abundance and virulence of the virus in the saliva as well as upon the susceptibility of the subject. Some animals are insusceptible, either naturally or by reason of their having been previously subjected to the action of the virus; yet under a full, virulent dose nearly all succumb. The theory that rabies kills more animals in summer than in winter has been weakened by statistics. Burrell has shown, according to the record of cases of rabies in his infirmary from 1859 to 1872, that they were not more frequent in summer than in winter. Three hundred and fourteen cases of rabies of the dog observed at the Alfort School during the years from 1887 to 1890 are divided as follows: January, February, and March, 130 cases; April, May, and June, 60 cases; July, August, and September, 50 cases; October, November, and December, 47 cases. Bouley records a greater number of cases in December, January, and February than in any other three months of the year. The real explanation of the greater prevalence in the spring and summer is found in the fact that bitches rut in the spring, and a number of the candidates for their favors bite each other fatally. This is aggravated by the fact that the generative instincts are stimulated in the early stages of rabies. This further explains the predominance of rabies in males. The irritable rabid dog antagonizes his male competitors and respects the female object of their common desire. There is, of course, only one cause of the disease, namely, inoculation from an animal suffering from the disease, although excitement will hasten the eruption in the inoculated animal.

It may be assumed that the virulent principle which causes the disease is an organic germ; but so far all attempts to isolate and cultivate it in pure culture have resulted in failure, and the microbe cannot yet be certainly identified.

Rabies agrees with all other germ diseases in that it develops only after inoculation, in that one attack usually fortifies the system against a second, and that in Australia, Tasmania, New Zealand, St. Helena, South Africa, and West Africa, from which mad dogs are excluded, it has never appeared, while in Buenos Ayres, Hong Kong, and Malta, where they have been allowed freedom it has become prevalent. What has never occurred in the past never need be looked for in the future. Cases in which infection is denied because the dog was shut up will be explained by a more thorough investigation. Rabid dogs will leap high fences to reach supposed enemies, and rabid rats and other vermin enter through small holes.

Rabies, like most microbic diseases, is at first confined to the region of the bite, and the tissues there are alone affected. When fully developed the infection is resident in the blood and all vascular tissues, yet the usual source of infection is through the bronchial mucus and the saliva, both of which are especially virulent and are naturally implanted by the teeth. This virulence is not confined to carnivora, but has been experimentally demonstrated in omnivora and herbivora. Various cases of infection from man to man are on record. Drying of saliva or blood, apart from heat or putrefaction, does not destroy its virulence. The knives fouled on rabid animals have been used for successful inoculation months and years later. Among other methods of infection besides the bite may be named the licking of sores by a dog in the early stages of rabies and the occupation of kennels or stalls that have previously harbored rabid animals. Rabies has been known to attack a second pack of hounds after the first pack had been killed out because of the disease. In one case a man was infected by using his teeth to untie a knot in a rope that had been used to tie a mad dog. Infection in man has been caused by a bite from a dog that had been previously fighting a rabid dog, and, again, from the scratch of a cat that had been licking its claws. In some cases of incipient rabies in dogs the saliva has been virulent before any outward symptoms were shown. Hence, all dogs, however sound in appearance, should be objects of suspicion in an infected district.

The anatomical alterations found in autopsies upon rabid animals are neither constant nor specific. Rabies is especially characterized by the absence of important organic lesions. There are emaciation; mucus about the eyes, nostrils, and prepuce; staring coat; venous congestion; the tongue has a dirty brown fur. In the dog foreign bodies, such as straw, hair, pieces of wood and clothing may be found in the mouth and pharynx; the stomach is congested and contracted, and contains little or no food, but a mixture of foreign bodies and indigestible substances which are highly characteristic of the disease. Wortley Axe in a total of 200 autopsies has found in 180 cases, or 90 per cent., absence of food and the presence of indigestible foreign bodies in the stomach. For him this latter fact is the most important from a diagnostic stand-point. Post-mortem diagnosis can be established with certainty only by inoculation; but when at the autopsy of a dog which manifested aggressive tendencies during the last period of life, or which had bitten animals or people, we recognize the ordinary symptoms of rabies, especially the presence of foreign bodies in the stomach, we must, without hesitation, affirm the case to be rabies, and proceed accordingly.

Babes describes changes in the nerve cells, with cloudiness of the protoplasm. These have been especially noticed in connection with the motor centres in the medulla oblongata, but also in the gray matter of the cerebrum. The nerve trunks, too, may be the seat of congestion and the fibres undergo a granular degeneration.

The lesions to be especially relied on in the dog are the congestion of the fauces and throat; the congested, infiltrated, or ulcerated state of the stomach; the absence of food; the presence of foreign bodies; some congestion of the small intestines; empty or nearly empty bladder; mucus or mucopurulent secretion oozing from various openings; congestion of superficial veins; congestion of the brain and the meninges. These, with the history of the cases, are usually sufficient to identify the disease. It should be added, however, that in the paralytic or lethargic form in the dog there may be an entire absence of foreign bodies in the stomach.

Of the 17 cases in the dog that I have met with in my practice in the past year most of them were of the paralytic form; 7 out of the 17 cases have been of the furious form; 10 of the animals were either known to have been bitten or had been exposed to rabid dogs. The remainder were not exposed to the disease, to the knowledge of the owners; yet it is possible that they were, not having been confined and having had access to the street at will. The period of incubation varies greatly. In inoculation with potent virus or street virus upon the brain it is six days. In other parts of the body it varies from 16 to 240 days, with an average of 25 days.

Rabies appears under two clinical forms, which are designated by the expression of furious rabies and mute rabies. Formerly these two forms were considered two distinct diseases, but this view was abandoned long ago.

According to Pasteur, furious rabies occurs when the brain is invaded by the rabid virus, and mute when it reaches the spinal cord first. His claim is that we may produce the former experimentally by directly depositing the virus on the surface of the brain, the latter by injecting it into subcutaneous connective tissue. I rather doubt the correctness of this assertion, as I am inclined to think that only a very small percentage of dogs are bitten on the face compared to the number bitten on other parts of the body. While in man the percentage of bites on the face is very small, the majority of cases of rabies in man are of the furious form. By depositing the virus directly on the brain it produces a disease within a very short period of incubation, which possibly accounts for the activity of the symptoms excited by this form of inoculation.

The prevention of rabies can be accomplished in cities and towns only by passing ordinances and compelling owners to muzzle their dogs when the outbreak occurs in a community. The animal should wear an efficient muzzle, as rabies is propagated in nature only by biting. Such a regulation, if strictly enforced, would stop the transmission of the disease and soon lead to its disappearance.

As the disease is just as prevalent in winter as in summer, the dogs should be muzzled the year round until the disease has made its entire disappearance. However, this is at once opposed by a class of citizens who hold it to be cruel and unnecessary.

Some muzzles are unquestionably cruel, but a properly made muzzle is not cruel, nor does it greatly inconvenience the dog after he has become accustomed to it. A certain kind of muzzle should be prescribed by the authorities. It should be one which covers the mouth with a wire cage, so as to prevent biting, but which does not interfere with the movements of the mouth and the ingestion of liquids. There are many who claim that dogs do not wear the muzzle at home, and that when they develop rabies and escape it is always when they are not muzzled. Admitting this argument to be true, nevertheless it is a fact that, if all dogs were required to be muzzled when in public, the appearance of a dog without a muzzle would at once attract attention, leading persons to avoid it and causing its early seizure by the authorities. Children might be taught to fear unmuzzled dogs and to keep at a distance. The results which have been attained by muzzles justify the enforcement of a muzzling ordinance whenever there is an outbreak. In Berlin, where a rigorous muzzle law was enforced, the disease was entirely eradicated; also in Great Britain the muzzle has been adopted with great success.

The treatment of the bite should receive the first attention. If possible, the wound should be cauterized by the actual cautery; if not, chloride of zinc, bichloride of mercury, caustic potash, silver nitrate, or sulphate of copper or iron should be used. Care should be taken to apply it thoroughly to all recesses of the wound. If mineral acids or other liquid caustics are employed they may be delivered into the minute recesses through a pipette or a plug of cotton wound around a stick or with a syringe. A delay of several hours or days is no warrant for omitting cauterization, for in man it has always a good moral effect in preventing hydrophobia, and it is also possible that the poison may remain for some time in the region of the sore.

Senn's advice is to excise the adjacent tissue. This may be followed, but not to the exclusion of a thorough disinfection.

When a person has been bitten by a dog with symptoms of rabies the dog should not be killed, but should be chained in a place where it will have no chance to do harm to anyone. There it should be kept until the disease has had a chance to thoroughly develop. If it dies from rabies, and the bite has not had the necessary treatment, the bitten person should at once take the now famous Pasteur cure. The Pasteur Institute at Chicago has been established eleven years, in which time 1262 patients have been treated. Of these only seven have died, making a death-rate of less than $\frac{1}{2}$ of 1 per cent. As a remedial agent for the bitten the Pasteur treatment is unquestionably effective, as is shown by the great percentage of cures.

### Discussion.

Dr. Lyford related a case of rabies in a horse. The horse was bitten on the nose by a dog, and, later on, on the hind limb while being driven through the street by its owner. The offending dog was killed. On the twentieth day the horse was reported by the owner to be acting strangely. Dr. Lyford visited him, and found him acting violently and showing a great tendency to bite everyone except his owner, who could handle him without difficulty. A diagnosis of rabies was made and the owner instructed to tie him with a chain. The next day the horse was much worse, and the tendency to bite was much more developed. The stall bore marks of the animal's teeth, and his mouth was injured and bleeding. A broom was held out to the horse, and he grasped it in his teeth and shook it as a dog would shake a rat, then lay down and rolled up on his back, still holding the broom firmly in his jaws. The horse was killed and the head sent to the University of Minnesota. Three rabbits were inoculated subdurally, and in due course of time developed rabies.

Dr. Repp described the microscopical changes detailed by Van Gehuchten and Nelis in Europe, and later by Ravenel and McCarthy in this country.

Dr. Brimhall reported a case in his experience in which it was proven by successive rabbit inoculations that the milk of a cow suffering from rabies was virulent and capable of producing rabies.

